# Prevalence, Characteristics, and Determinants of Suboptimal Care in the Initial Management of Community-Onset Severe Bacterial Infections in Children

**DOI:** 10.1001/jamanetworkopen.2022.16778

**Published:** 2022-06-13

**Authors:** Fleur Lorton, Martin Chalumeau, Alain Martinot, Rémy Assathiany, Jean-Michel Roué, Pierre Bourgoin, Julie Chantreuil, Gérald Boussicault, Théophile Gaillot, Jean-Pascal Saulnier, Jocelyne Caillon, Christèle Gras-Le Guen, Elise Launay

**Affiliations:** 1 Centre of Clinical Research Femme Enfant Adolescent, Hôpital Femme Enfant Adolescent, Inserm 1413, CHU de Nantes, Nantes, France; 2 Inserm UMR 1153, Obstetrical, Perinatal and Pediatric Epidemiology Research Team (Epopé), Centre of Research in Epidemiology and Statistics, Université Paris Cité, Paris, France; 3 Department of Pediatrics and Pediatric Emergency, Hôpital Femme Enfant Adolescent, CHU de Nantes, Nantes, France; 4 Department of General Pediatrics and Pediatric Infectious Diseases, Necker Hospital for Sick Children, Assistance Publique-Hôpitaux de Paris, Université Paris Cité, Paris, France; 5 Univ Lille, ULR 2694-METRICS, Evaluation des technologies de Santé et des pratiques médicales, CHU Lille, Lille, France; 6 Association pour la Recherche et l’Enseignement en Pédiatrie Générale, Association Française de Pédiatrie Ambulatoire, Cabinet de Pédiatrie, Issy-les-Moulineaux, France; 7 Department of Pediatric and Neonatal Critical Care, Brest University Hospital, Brest, France; 8 Department of Pediatric and Neonatal Critical Care, Hôpital Femme Enfant Adolescent, CHU de Nantes, Nantes, France; 9 Department of Pediatric and Neonatal Critical Care, Hôpital Clocheville, CHU de Tours, Tours, France; 10 Department of Pediatric Critical Care, CHU d’Angers, Angers, France; 11 Department of Pediatric Critical Care, Hôpital Sud, CHU de Rennes, Rennes, France; 12 Department of Pediatric and Neonatal Critical Care, Tour Jean Bernard, CHU de Poitiers, Poitiers, France; 13 Department of Microbiology, Hôtel Dieu, CHU de Nantes, Nantes, France

## Abstract

**Importance:**

Assessment of the quality of initial care is necessary to target priority actions that can reduce the still high morbidity and mortality due to community-onset severe bacterial infections (COSBIs) among children.

**Objective:**

To study the prevalence, characteristics, and determinants of suboptimal care in the initial management of COSBIs.

**Design, Setting, and Participants:**

This prospective, population-based, cohort study and confidential enquiry was conducted between August 2009 and January 2014 in western France, a region accounting for 15% of the French pediatric population (1 968 474 children aged 1 month to 16 years) and including 6 pediatric intensive care units (PICUs) and 35 emergency departments. Participants included all children aged 1 month to 16 years who died before PICU admission or were admitted to a PICU with a COSBI (ie, bacterial sepsis, including meningitis, purpura fulminans, and pulmonary, osteoarticular, intra-abdominal, cardiac, and soft-tissue severe infections). Data were analyzed from March to June 2020.

**Exposures:**

Suboptimal care determined according to evaluation of 8 types of care: (1) the delay in seeking care by family, (2) the physician’s evaluation of severity, (3) the patient’s referral at the first consultation with signs of severity, (4) the timing and (5) dosage of antibiotic treatment, (6) the timing and (7) volume of fluid bolus administration, and (8) the clinical reassessment after fluid bolus.

**Main Outcomes and Measures:**

Two experts assessed the quality of care before death or PICU admission as optimal, possibly suboptimal, or certainly suboptimal. The consequences and determinants of certainly suboptimal care were identified with multinomial logistic regression and generalized linear mixed models.

**Results:**

Of the 259 children included (median [IQR] age, 24 [6-66] months; 143 boys [55.2%]), 27 (10.4%) died, and 25 (9.6%) had severe sequelae at PICU discharge. The quality of care was certainly suboptimal in 89 cases (34.4%). Suboptimal care was more frequent in children with sequelae (adjusted odds ratio [aOR], 5.61; 95% CI, 1.19-26.36) and less frequent in children who died (aOR, 0.16; 95% CI, 0.04-0.65) vs those surviving without sequelae. Factors independently associated with suboptimal care were age younger than 5 years (aOR, 3.15; 95% CI, 1.25-7.90), diagnosis of sepsis with no source (aOR, 5.77; 95% CI, 1.64-20.30) or meningitis (aOR, 3.39; 95% CI, 1.15-9.96) vs other severe infections, and care by a primary care physician (aOR, 3.22; 95% CI, 1.17-8.88) vs a pediatric hospital service.

**Conclusions and Relevance:**

This study found that suboptimal care is frequent in the initial management of COSBI and is associated with severe sequelae. The paradoxical association with reduced risk of death may be explained by an insufficient adjustment on bacterial or host intrinsic factors. Management could be optimized by improving the quality of primary care, especially for young children.

## Key Points

**Question:**

What are the prevalence, characteristics, and determinants of suboptimal care in the initial management of community-onset severe bacterial infections in children?

**Findings:**

In this cohort study that included 259 children, suboptimal care before pediatric intensive care unit admission was frequent and was associated with severe sequelae in children with a community-onset severe bacterial infection. The youngest children and those initially cared for by a primary care physician were at increased risk of suboptimal care.

**Meaning:**

These findings suggest that medical care could be optimized, especially for young children, by improving the preparedness of primary care physicians.

## Teaser

This cohort study examines the prevalence, characteristics, and determinants of suboptimal care in the initial management of community-onset severe bacterial infection in children in western France.

## Introduction

In 2017, the World Health Organization adopted a resolution to improve the prevention, diagnosis, and management of sepsis and reduce its burden.^1,2^ In high-income countries, severe bacterial infections (SBIs) are a major cause of morbidity and mortality in children,^3^ accounting for approximately 25% of deaths in pediatric intensive care units (PICUs).^4^ Approximately 30% of survivors experience severe sequelae, such as amputation, neurodevelopmental impairment, or hearing loss.^5,6,7^ To prevent the adverse progression of an infection and reduce its morbidity and mortality,^8^ optimal management is necessary and relies on 3 crucial steps^9,10,11,12,13,14^: first, rapid seeking of care by the family as soon as signs of severity appear; second, recognition of the severity of the infection by the physician and appropriate referral; and third, treatment in accordance with the international guidelines issued by the Surviving Sepsis Campaign.^15,16,17,18,19^ Several authors have hypothesized that the current remaining mortality of SBIs is related to suboptimal care in 1 or more of these steps.^20,21,22^

Available data on the quality of the initial management of SBIs show that 75% to 92% of children who died received suboptimal care before admission to a PICU,^21,22,23^ notably a delay in seeking medical care, an underestimation of the severity by the physician, or a delay in treatment.^23,24,25^ Suboptimal care was found to be associated with death or sequelae.^13,20,21,22^ Determinants of suboptimal care include age younger than 1 year and nonspecialization in pediatrics by the physician caring for the child.^20,22^ However, data sources are limited by their single-center,^21,24^ retrospective,^13,20,21,22,23,25^ and/or hospital-based^21,24,25^ designs; only studying 1 bacterium^13,22,24^; or restricting the analyses to the association between suboptimal care and death but not severe sequelae.^20,21,22^

To our knowledge, no prospective, large-scale, population-based study of this topic has been performed, but accurate data on the quality of the initial care provided are necessary to target priority actions for improving the survival of children without sequelae, as recommended by the World Health Organization.^26^ The objective of the present study was to describe the prevalence, characteristics, and determinants of suboptimal care during the initial management of an SBI in children in a large, prospective, population-based confidential enquiry.

## Methods

We conducted this cohort study and confidential enquiry in western France, a region accounting for 15% of the French pediatric population, with 1 968 474 children aged 1 month to 16 years during the study period.^27^ The study was approved by the local ethics committee (Comité de Protection des Personnes Ouest II-Angers), was registered at ClinicalTrials.gov (NCT01485705), and was reported according to the Strengthening the Reporting of Observational Studies in Epidemiology (STROBE) reporting guidelines. Oral consent was obtained from study participants and their parents.

### Study Design and Participants

As previously described in detail,^28,29^ we included all children from age 1 month to 16 years who died before PICU admission or were admitted to a PICU in the context of a community-onset SBI (COSBI) between August 2009 and January 2014. SBI was defined as bacterial sepsis (according to the definition of the International Consensus Conference on Children’s Sepsis^15^) requiring hospitalization in a PICU or resulting in death before hospitalization.^20,23^ The community-onset nature of the infection was defined by symptoms occurring at home or within the first 48 hours of hospitalization.^30^ The outcome of the children was classified as survival without immediate severe sequelae, survival with sequelae at PICU discharge (paresis, paralysis, ataxia, neuropathic pain, sensory deficit including hearing loss, hypotonia, hydrocephaly, epilepsy, necrotic skin lesions requiring skin grafting, localized necrotic skin lesions on the face or hands, amputation, or cardiac, kidney, cerebral, hepatic, or pulmonary failure),^31,32^ or death. Only children for whom the quality of the global management could be assessed were included in the analysis.

The study area included 6 university hospitals with a PICU and 35 hospitals with an emergency department specialized or not in pediatrics. The organization of care in this geographical area provided that children requiring intensive care for a severe infection should be transferred to 1 of these 6 PICUs. A medical investigator (J.M.R., P.B., J.C., G.B., T.G., and J.P.S.) in each participating PICU prospectively screened all patients admitted with an infection. The exhaustiveness of inclusions was checked in the administrative databases. Deaths at home in a context of fever were identified by interventions of medical emergency mobile units and the reference centers for unexpected infant death.^33^ Given the organization of care in France, a child who died at home or in a hospital ward other than a PICU would have been registered in 1 of those units. In addition, all children younger than 2 years who die at home usually have bacteriological samples taken during the subsequent autopsy performed in the hospital, which allows for identifying cases of bacterial infection not in a context of fever.

### Data Collection

We collected data on (1) the characteristics of the children (age, sex, and comorbidities), (2) the characteristics of the infection (symptoms, presence of early signs of severity, and final diagnosis), (3) the outcome of the children, (4) the characteristics of the health care provision (density of medical doctors [MDs] in the home department, classified as low [230-280 MDs per 100 000 population], intermediate [281-320 MDs per 100 000 population], or high [321-370 MDs per 100 000 population]),^27^ and (5) the characteristics of the initial care pathway for each child from the onset of symptoms to PICU admission. Data were collected by directly questioning parents and caregivers and from medical records.

### Quality of Care Evaluation

For each child, a case summary was prepared that included the child’s characteristics, date and time of onset of each symptom, and details of each medical consultation and was reviewed independently by 2 experts (1 private pediatrician [R.A.] and 1 pediatric critical care physician [A.M.]) not involved in the care of the child. According to the methods for confidential enquiries,^34^ the experts knew that the children had presented with a serious event (ie, hospitalization in a PICU for a severe bacterial infection or death before admission), but they were blinded to the final diagnosis and patient outcome (death or survival with or without severe sequelae). The experts evaluated the quality of the global management according to a precise evaluation of 8 pivotal types of care selected from a literature review,^22,23,24,35^ which all can be theoretically improved by corrective actions: (1) the delay in seeking care by family, (2) the physician’s evaluation of severity, (3) the patient’s referral at the first consultation with signs of severity, (4) the timing and (5) dosage of antibiotic treatment, (6) the timing and (7) volume of fluid bolus administration, and (8) the clinical reassessment after fluid bolus. Four categories were proposed to the experts to evaluate the quality of the global management and each of the 8 major types of care: optimal, possibly suboptimal, certainly suboptimal, and cannot judge. The experts were asked to base their analyses on current national and international guidelines^16^ when existing (eg, time to antibiotics in case of sepsis and antibiotic and fluid bolus dosage) or on their own expertise when no formal consensus existed (eg, time to consultation). The degree of agreement among experts was assessed by Cohen κ coefficient^36^ and interpreted according to the scale proposed by Landis and Koch.^37^ In case of a discrepancy between the experts’ judgment, the case summary was reviewed by a third expert (E.L.).

### Statistical Analysis

The frequency of suboptimal care was calculated for the global management and for each of the 8 major types of care in the entire population and according to the outcome of children. To analyze the adjusted association between the quality of care and the outcome, we used a multinomial logistic regression model, with survival without severe sequelae as the reference. The variables selected in the final model were the quality of the global management, the risk factors for child mortality from SBIs already established in the literature^20,22^ and potential confounding factors. We constructed a directed acyclic graph^38,39^ to represent the relationship between the quality of initial care, the potential confounding factors, and the child outcome (eFigure in the Supplement). To analyze the determinants of the quality of care, we used a generalized linear mixed model with a random effect on the PICU. The intraclass correlation coefficient was estimated to evaluate the proportion of total variance in the probability of suboptimal care attributable to the variance between centers.^40,41,42^ We restricted the potential association analyses involving quality of care to only children whose care was judged optimal or certainly suboptimal to gain contrast.

The number of patients to be recruited was based on the measure of the association between suboptimal care and the occurrence of death in children with a COSBI (eAppendix in the Supplement). We used a significance level of *P* < .05 and 2-tailed hypothesis tests. Statistical analyses were conducted from March to June 2020 involved using R statistical software version 4.0.0 (R Project for Statistical Computing).

## Results

### Patients and Care Pathway

During the study period, 261 children had a COSBI in western France and 259 children were included in the analyses (Figure). The median (IQR) age at diagnosis was 24 (6-66) months, and 143 children (55.2%) were boys (male-to-female sex ratio, 1.3) (Table 1). Of the 259 children, 63 (24.3%; 95% CI, 19.2%-30.0%) had 1 or more known comorbidities at the time of the COSBI, mainly a history of severe infection (28 children), congenital malformations or neurological disease (14 children), and prematurity (11 children). Overall, 27 children (10.4%; 95% CI, 7.0%-14.8%) died of the COSBI, 3 before admission to hospital, and 25 (9.6%; 95% CI, 6.0%-13.9%) had severe sequelae at PICU discharge (Table 1). The most frequent diagnoses were meningitis (84 children [32.4%]), purpura fulminans (59 children [22.8%]), and sepsis or septic shock with no identified source (43 children [16.6%]) (Table 1). In 71 cases (27.4%; 95% CI, 22.1%-33.3%), the children had hemodynamic severity signs at the first medical consultation (Table 1).

**Figure.**
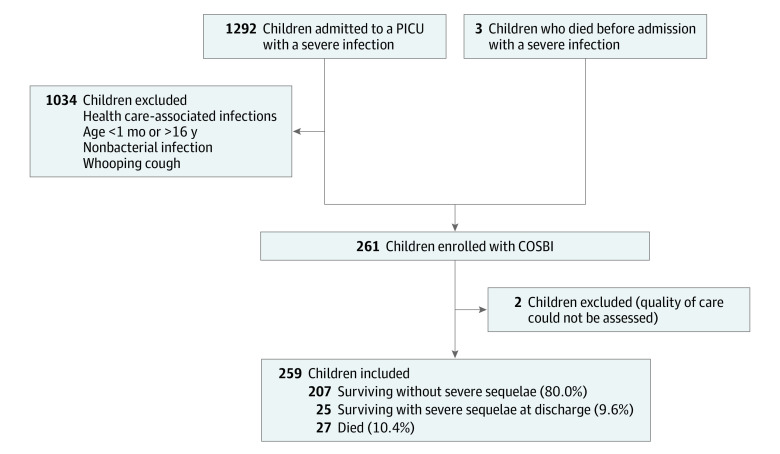
Flowchart of Children With a Community-Onset Severe Bacterial Infection (COSBI) Included in the Study PICU indicates pediatric intensive care unit.

**Table 1.** Characteristics of Children With a Community-Onset Severe Bacterial Infection and Their Association With Outcome on Bivariable Analysis

Characteristic	Patients, No. (%)				Bivariable analyses			
	Total (N = 259)	Surviving without sequelae (n = 207)	Died (n = 27)	Surviving with sequelae (n = 25)	Surviving without sequelae vs died		Surviving without sequelae vs surviving with sequelae	
					OR (95% CI)	*P* value	OR (95% CI)	*P* value
Age								
Median (IQR), mo	24 (6-66)	28 (7-72)	19 (5-35)	13 (5-27)	NA	NA	NA	NA
1 mo to 5 y	185 (71.4)	141 (68.1)	23 (85.2)	21 (84.0)	2.69 (0.89-8.10)	.08	2.46 (0.81-7.44)	.11
≥5 y	74 (28.6)	66 (31.9)	4 (14.8)	4 (16.0)	1 [Reference]		1 [Reference]	
Sex								
Female	116 (44.8)	92 (44.4)	15 (55.6)	9 (36.0)	1.56 (0.70-3.50)	.28	0.70 (0.30-1.66)	.42
Male	143 (55.2)	115 (55.6)	12 (44.4)	16 (64.0)	1 [Reference]		1 [Reference]	
Comorbidities								
Yes	63 (24.3)	45 (21.7)	12 (44.4)	6 (24.0)	2.88 (1.26-6.59)	.01	1.14 (0.43-3.01)	.80
No	196 (75.7)	162 (78.3)	15 (55.6)	19 (76.0)	1 [Reference]		1 [Reference]	
Hemodynamic severity signs at first consultation								
Yes	71 (27.4)	51 (24.6)	14 (51.9)	6 (24.0)	3.29 (1.45-7.47)	.004	0.97 (0.37-2.55)	.94
No	188 (72.6)	156 (75.4)	13 (48.1)	19 (76.0)	1 [Reference]		1 [Reference]	
Discharge diagnosis								
Meningitis	84 (32.4)	66 (31.9)	8 (29.6)	10 (40.0)	1.94 (0.56-6.76)	.30	1.94 (0.63-5.99)	.25
Purpura fulminans	59 (22.8)	44 (21.3)	9 (33.4)	6 (24.0)	3.27 (0.95-11.30)	.06	1.75 (0.50-6.08)	.38
Sepsis with no source	43 (16.6)	33 (15.9)	6 (22.2)	4 (16.0)	2.91 (0.77-11.03)	.12	1.55 (0.39-6.17)	.53
Other^a^	73 (28.2)	64 (30.9)	4 (14.8)	5 (20.0)	1 [Reference]	NA	1 [Reference]	NA

Abbreviations: NA, not applicable; OR, odds ratio.

^a^ Other diagnosis includes pulmonary, urinary, osteoarticular, intra-abdominal, cardiac, and soft-tissue severe infections.

The first medical service in the care pathway was with a primary care physician in 147 cases (56.8%), in an emergency department in 95 cases (36.7%), or with a medical emergency mobile unit in 17 cases (6.5%). The total number of medical consultations before PICU admission per child ranged from 1 to 8 (median [IQR], 3 [2-4] consultations).

### Quality of Care

The degree of agreement among experts assessing the quality of care for the global management was considered substantial according to the Landis and Koch scale (eTable 1 in the Supplement).^37^ The number of suboptimal types of care per child ranged from 0 to 6 (median [IQR], 2 [1-3] types of care) (Table 2). The quality of global management before admission to a PICU was considered optimal in 70 cases overall (27.0%; 95% CI, 21.7%-32.9%), certainly suboptimal in 89 cases (34.4%; 95% CI, 28.6%-40.5%), and possibly suboptimal in 100 cases (38.6%; 95% CI, 32.6%-44.8%) (eTable 2 in the Supplement). Of the 25 children with severe sequelae, 15 (60.0%; 95% CI, 38.7%-78.9%) had certainly suboptimal management. Of the 8 major types of care evaluated, the timing of antibiotic therapy (133 cases [51.6%]) and fluid bolus (128 cases [55.7%]) were the 2 most frequent suboptimal types of care (eTable 2 in the Supplement). Timing to seek care by family and the initial severity assessment by physician was considered optimal in most cases (184 cases [71.0%] and 186 cases [71.8%], respectively). Examples of the different categories of quality of care for the 8 major types of care are reported in eTable 3 in the Supplement.

**Table 2.** Characteristics of the Quality of Care Before Admission to a Pediatric Intensive Care Unit in Children With a Community-Onset Severe Bacterial Infection and Their Association With Outcome on Bivariable Analysis

Variable	Patients, No. (%)				Bivariable analyses			
	Total (N = 259)	Surviving without sequelae (n = 207)	Died (n = 27)	Surviving with sequelae (n = 25)	Surviving without sequelae vs died		Surviving without sequelae vs surviving with sequelae	
					OR (95% CI)	*P* value	OR (95% CI)	*P* value
Suboptimal types of cares, median (IQR), No./child	2 (1-3)	2 (1-3)	1 (0-2)	2 (2-3)	0.46 (0.31-0.68)	<.001	1.05 (0.78-1.42)	.73
Global management								
Children, No.^a^	159	127	15	17	NA	NA	NA	NA
Certainly suboptimal	89 (56.0)	71 (55.9)	3 (20.0)	15 (88.2)	0.20 (0.05-0.73)	.02	5.91 (1.30-26.94)	.02
Optimal	70 (44.0)	56 (44.1)	12 (80.0)	2 (11.8)	1 [Reference]		1 [Reference]	
Time in seeking care								
Children, No.	247	195	27	25	NA	NA	NA	NA
Certainly suboptimal	63 (25.5)	51 (26.2)	6 (22.2)	6 (24.0)	0.81 (0.31-2.11)	.66	0.89 (0.34-2.36)	.82
Optimal	184 (72.5)	144 (73.8)	21 (77.8)	19 (76.0)	1 [Reference]		1 [Reference]	
Evaluation of severity								
Children, No.	235	189	23	23	NA	NA	NA	NA
Certainly suboptimal	49 (20.9)	40 (21.2)	3 (13.0)	6 (26.1)	0.56 (0.16-1.98)	.37	1.31 (0.49-3.55)	.59
Optimal	186 (79.1)	149 (78.8)	20 (87.0)	17 (73.9)	1 [Reference]		1 [Reference]	
Patient referral								
Children, No.	231	186	23	22	NA	NA	NA	NA
Certainly suboptimal	48 (20.8)	39 (21.0)	2 (8.7)	7 (31.8)	0.36 (0.08-1.60)	.18	1.76 (0.67-4.61)	.25
Optimal	183 (79.2)	147 (79.0)	21 (91.3)	15 (68.2)	1 [Reference]		1 [Reference]	
Antibiotic therapy timing								
Children, No.	253	203	25	25	NA	NA	NA	NA
Certainly suboptimal	133 (52.6)	110 (54.2)	7 (28.0)	16 (64.0)	0.33 (0.13-0.82)	.02	1.50 (0.63-3.56)	.35
Optimal	120 (47.4)	93 (45.8)	18 (72.0)	9 (36.0)	1 [Reference]		1 [Reference]	
Antibiotic therapy dosage								
Children, No.	214	175	21	18	NA	NA	NA	NA
Certainly suboptimal	25 (11.7)	22 (12.6)	1 (4.8)	2 (11.1)	0.35 (0.04-2.72)	.31	0.90 (0.19-4.04)	.86
Optimal	189 (88.3)	153 (87.4)	20 (95.2)	16 (88.9)	1 [Reference]		1 [Reference]	
Fluid bolus timing								
Children, No.	225	182	22	21	NA	NA	NA	NA
Certainly suboptimal	128 (56.9)	110 (60.4)	6 (27.3)	12 (57.1)	0.25 (0.09-0.66)	.005	0.87 (0.35-2.18)	.77
Optimal	97 (43.1)	72 (39.6)	16 (72.7)	9 (42.9)	1 [Reference]		1 [Reference]	
Fluid bolus volume								
Children, No.	153	117	21	15	NA	NA	NA	NA
Total received volume, median (IQR), mL/kg	35 (20-50)	33 (20-50)	40 (23-43)	40 (20-40)	NA	NA	NA	NA
Certainly suboptimal	56 (36.6)	49 (41.9)	3 (14.3)	4 (26.7)	0.23 (0.06-0.83)	.02	0.50 (0.15-1.68)	.26
Optimal	97 (63.4)	68 (58.1)	18 (85.7)	11 (73.3)	1 [Reference]		1 [Reference]	
Assessment after fluid bolus								
Children, No.	152	120	19	13	NA	NA	NA	NA
Certainly suboptimal	29 (19.1)	23 (19.2)	2 (10.5)	4 (30.8)	0.50 (0.11-2.30)	.37	1.87 (0.53-6.62)	.33
Optimal	123 (80.9)	97 (80.8)	17 (89.5)	9 (69.2)	1 [Reference]		1 [Reference]	

Abbreviations: NA, not applicable; OR, odds ratio.

^a^ Refers to children affected by the care assessed and whose quality of care was assessed as certainly suboptimal or optimal; the possibly suboptimal category was excluded from these analyses.

### Factors Associated With Poor Outcome

Compared with survival without sequelae, death was associated with the presence of comorbidities (odds ratio [OR], 2.88; 95% CI, 1.26-6.59) or hemodynamic signs of severity at the first medical consultation (OR, 3.29; 95% CI, 1.45-7.47) (Table 1). Among children who died, the median (IQR) number of consultations per child was 2 (2-3), and the median (IQR) number of care opportunities (ie, the total number of types of care performed or that should have been performed) was 5 (5-5) and was lower than among children who survived without sequelae (consultations, OR, 0.68; 95% CI, 0.48-0.96; care opportunities, OR, 0.58; 95% CI, 0.35-0.97). The median number of consultations and care opportunities did not differ between children surviving without sequelae and those with sequelae (consultations, OR, 1.06; 95% CI, 0.79-1.43; care opportunities, OR, 1.10; 95% CI, 0.76-1.58). Death was less frequent in children with suboptimal antibiotic therapy timing (OR, 0.33; 95% CI, 0.13-0.82) and suboptimal fluid bolus timing (OR, 0.25; 95% CI, 0.09-0.66) and volume (OR, 0.23; 95% CI, 0.06-0.83) than in children surviving without sequelae (Table 2).

After adjustment for child’s age, presence of comorbidities, presence of hemodynamic severity signs at first consultation, and discharge diagnosis, suboptimal global management was significantly associated with the child’s outcome (adjusted OR [aOR], 0.16 [95% CI, 0.04-0.65] for children who died and 5.61 [95% CI, 1.19-26.36] for children with sequelae, both compared with children who survived without sequelae) (Table 3). The presence of comorbidities was independently associated with death (aOR, 4.49; 95% CI, 1.25-16.09) (Table 3).

**Table 3.** Multivariable Analysis of the Adjusted Association Between the Quality of the Global Management Before Admission to a Pediatric Intensive Care Unit in Children With a Community-Onset Severe Bacterial Infection and the Outcome

Variable	Patients, No. (%)^a^				Multivariable analysis			
	Total (N = 159)	Surviving without sequelae (n = 127)	Died (n = 15)	Surviving with sequelae (n = 17)	Surviving without sequelae vs died		Surviving without sequelae vs surviving with sequelae	
					aOR (95% CI)	*P* value	aOR (95% CI)	*P* value
Age								
1 mo to 5 y	113 (71.1)	87 (68.5)	12 (80.0)	14 (82.4)	2.38 (0.55-10.31)	.25	1.92 (0.46-8.09)	.37
≥5 y	46 (28.9)	40 (31.5)	3 (20.0)	3 (17.6)	1 [Reference]		1 [Reference]	
Comorbidities								
Yes	39 (24.5)	27 (21.3)	7 (46.7)	5 (29.4)	4.49 (1.25-16.09)	.02	1.65 (0.42-6.43)	.47
No	120 (75.5)	100 (78.7)	8 (53.3)	12 (70.6)	1 [Reference]		1 [Reference]	
Hemodynamic severity signs at first consultation								
Yes	49 (30.8)	35 (27.6)	8 (53.3)	6 (35.3)	1.54 (0.43-5.50)	.51	1.36 (0.40-4.58)	.62
No	110 (69.2)	92 (72.4)	7 (46.7)	11 (64.7)	1 [Reference]		1 [Reference]	
Discharge diagnosis								
Meningitis	52 (32.7)	42 (33.1)	4 (26.7)	6 (35.3)	1.91 (0.29-12.54)	.50	1.10 (0.24-5.07)	.90
Purpura fulminans	40 (25.2)	30 (23.6)	5 (33.3)	5 (29.4)	2.65 (0.35-19.77)	.34	1.61 (0.31-8.41)	.57
Sepsis with no source	29 (18.2)	22 (17.3)	4 (26.7)	3 (17.6)	3.42 (0.48-24.31)	.22	1.26 (0.22-7.34)	.80
Other^b^	38 (23.9)	33 (26.0)	2 (13.3)	3 (17.6)	1 [Reference]	NA	1 [Reference]	NA
Global management								
Certainly suboptimal	89 (56.0)	71 (55.9)	3 (20.0)	15 (88.2)	0.16 (0.04-0.65)	.01	5.61 (1.19-26.36)	.03
Optimal	70 (44.0)	56 (44.1)	12 (80.0)	2 (11.8)	1 [Reference]		1 [Reference]	

Abbreviation: aOR, adjusted odds ratio.

^a^ Only children whose global management was assessed optimal or certainly suboptimal were included in these analyses.

^b^ Other diagnosis includes pulmonary, urinary, osteoarticular, intra-abdominal, cardiac, and soft-tissue severe infections.

### Determinants of the Quality of Care

The potential determinants of the quality of care analyzed were child’s age, presence of comorbidity, presence of hemodynamic severity signs at first consultation, discharge diagnosis, density of MDs, and type of medical service visited at first consultation with signs of severity. On bivariable analysis, certainly suboptimal global management was associated with living in an area with a low (OR, 2.48; 95% CI, 1.17-5.29) or intermediate (OR, 2.29; 95% CI, 1.01-5.19) density of MDs or seeing a primary care physician for the first consultation with signs of severity (OR, 2.96; 95% CI, 1.19-7.37). After adjustment, certainly suboptimal global management (vs optimal) remained associated with consultation with a primary care physician (aOR, 3.22; 95% CI, 1.17-8.88) (Table 4). Suboptimal global management was associated with young age (aOR, 3.15; 95% CI, 1.25-7.90 for children younger than 5 years vs older children) and sepsis with no identified source (aOR, 5.77; 95% CI, 1.64-20.30) or meningitis (aOR, 3.39; 95% CI, 1.15-9.96) (vs other diagnosis: pulmonary, urinary, osteoarticular, intra-abdominal, cardiac, and soft-tissue severe infections). The intraclass correlation coefficient for the quality of care was estimated at 0.09 (95% CI, 0.01-0.44) (ie, 9% of the overall variance was attributable to the between-PICU variance).

**Table 4.** Multivariable Analysis of Potential Determinants of the Quality of the Global Management Before Admission to a Pediatric Intensive Care Unit in Children With a Community-Onset Severe Bacterial Infection

Variable	Patients, No. (%)			Multivariable analysis: optimal vs certainly suboptimal	
	Total (N = 159)	Optimal global management (n = 70)	Certainly suboptimal global management (n = 89)	aOR (95% CI)	*P* value
Patient variables					
Age					
1 mo to 5 y	113 (71.1)	45 (64.3)	68 (76.4)	3.15 (1.25-7.90)	.02
≥5 y	46 (28.9)	25 (35.7)	21 (23.6)	1 [Reference]	
Comorbidities					
Yes	39 (24.5)	16 (22.9)	23 (25.8)	2.53 (0.99-6.49)	.05
No	120 (75.5)	54 (77.1)	66 (74.2)	1 [Reference]	
Infection variables					
Hemodynamic severity signs at first consultation					
Yes	49 (30.8)	26 (37.1)	23 (25.8)	0.44 (0.18-1.09)	.08
No	110 (69.2)	44 (62.9)	66 (74.2)	1 [Reference]	
Discharge diagnosis					
Meningitis	52 (32.7)	19 (27.1)	33 (37.1)	3.39 (1.15-9.96)	.03
Purpura fulminans	40 (25.2)	19 (27.1)	21 (23.6)	2.49 (0.72-8.57)	.15
Sepsis with no source	29 (18.2)	12 (17.1)	17 (19.1)	5.77 (1.64-20.30)	.006
Other^a^	38 (23.9)	20 (28.6)	18 (20.2)	1 [Reference]	NA
Health care provision					
Density of medical doctors					
Low	48 (30.2)	16 (22.9)	32 (36.0)	2.44 (0.90-6.63)	.08
Intermediate	37 (23.3)	13 (18.6)	24 (27.0)	1.87 (0.58-5.98)	.30
High	74 (46.5)	41 (58.6)	33 (37.0)	1 [Reference]	NA
Medical service, No.^b^	154	68	86	NA	NA
Primary care physician	35 (22.7)	9 (13.2)	26 (30.2)	3.22 (1.17-8.88)	.02
Adult hospital department	27 (17.5)	16 (23.5)	11 (12.8)	0.61 (0.21-1.74)	.40
Pediatric hospital department	92 (59.7)	43 (63.2)	49 (57.0)	1 [Reference]	NA

Abbreviations: aOR, adjusted odds ratio; NA, not applicable.

^a^ Other diagnosis includes pulmonary, urinary, osteoarticular, intra-abdominal, cardiac, and soft tissue severe infections.

^b^ Refers to the first one who cared for the child with severity signs.

## Discussion

To our knowledge, this is the first large-scale, population-based, prospective cohort study and confidential enquiry of quality of care before PICU admission of children with a COSBI. Suboptimal care occurred frequently and was associated with severe sequelae vs survival without sequelae. We also identified several factors associated with increased risk of receiving suboptimal care: age younger than 5 years, a diagnosis of meningitis or sepsis with no identified source (vs other severe infections), and initial management by a primary care physician.

The frequency of suboptimal initial care was 34.4% in all children with a COSBI and 60.0% in children with severe sequelae. Comparing these results with previously reported frequencies of suboptimal care between 20% and 70% in children with an SBI is difficult because the care assessed and the definitions of suboptimal care vary among studies.^13,21,22,23,24^ The most common suboptimal types of care were inadequate timing for antibiotic therapy (51.6%) and fluid bolus (55.7%), although timing to seek care by family and the initial severity assessment by physician were considered optimal in most cases (71.0% and 71.8%, respectively). This issue of delays in treatment was highlighted in other studies in which most children with severe sepsis or septic shock did not receive antibiotic therapy^12,43^ or fluid bolus^14,21,44^ within the recommended time.

Suboptimal global management was independently associated with severe sequelae. This association seems consistent with data from the literature showing a decrease in SBI morbidity with early detection and appropriate management.^13,45^ The median number of consultations and opportunities for care per child was comparable to that of survivors without sequelae, which indirectly reflects a similar natural history of the disease. However, we did not find any association between a particular care intervention and the occurrence of sequelae, which could mean that the adverse outcome was related more to a cascade of failures in the care pathway for these children than to a specific care intervention.^46^

An unexpected finding of our study was that suboptimal global management was less common for children who died than for survivors without sequelae. Although most studies found an association between suboptimal care and death,^13,20,21,22^ a paradoxical association between optimal care and fatal outcome was previously reported, particularly in invasive *Neisseria meningitidis* infections such as purpura fulminans.^47,48^ Children who received on-time antibiotic therapy had more clinical signs of severity at admission^47^ and were probably those with the worst prognosis.^49,50^ Insufficient consideration of disease severity (bacterial or individual host genetic factors)^51,52,53^ and fulminant course is probably the source of residual confounding in our analyses, which explains our results on the association between increased survival and the suboptimality of global management and delayed time to antibiotic therapy and fluid bolus. In these children, the intrinsic risk of death once the infection has been contracted does not seem to be modified by the quality of care. Primary prevention actions, notably vaccination, would probably be the most effective in avoiding this fatal outcome. Unfortunately, we had demonstrated that lack of on-time vaccination was highly prevalent in this population.^28^ Finally, an important point of this finding was that the primary outcome of future studies assessing the impact of care on child outcome should not be a composite of mortality and morbidity because this could lead to the loss of any significant association.^5,54^

Analyses of the determinants of the quality of care allowed us to identify several possible targets for corrective action. The youngest children were at increased risk of suboptimal management, as previously shown.^20,55^ This age group often presents with nonspecific symptoms, so the practitioner cannot easily distinguish between a common viral infection and an SBI, which can lead to diagnostic or therapeutic delays.^56,57^ Some diagnoses, such as meningitis and sepsis, were also associated with risk of suboptimal care. A qualitative study of British general practitioners’ attitudes to suspected invasive bacterial infections reported that although known, these diagnoses were rarely raised in the presence of a febrile child because they were considered uncommon.^58^ In our study, the risk of receiving suboptimal care varied by the type of medical service first used, with increased risk associated with consultation with a primary care physician. Previous studies^20,59^ had shown increased frequency of suboptimal care without management by a pediatric specialist doctor. These deviations from optimal care may be due to a lack of expertise on the part of primary care practitioners, who are rarely exposed to these critical situations, or to difficulties in rapidly referring the child to the appropriate structures with a technical platform capable of delivering care that cannot be performed in a general practice, such as fluid bolus.

Our results may have several implications for improving the management of pediatric sepsis, especially in the prehospital setting. Early recognition should allow for its rapid management, thus avoiding missed opportunities and diagnostic errors. In the general population, parents’ lack of knowledge on certain symptoms of severity can lead to a delay in seeking care. Education campaigns on the signs requiring urgent medical attention in a febrile child could be beneficial for the general public. Such efforts are already under way in the United Kingdom and Ireland by the Meningitis Research Foundation, which uses pictograms or photographs of, for example, purpura, to publicize the signs that may suggest meningitis or sepsis.^60^ The red flags, which are symptoms or clinical signs associated with the presence of SBI that should be systematically searched in any febrile child (eg, cyanosis, polypnea, peripheral hypoperfusion, consciousness disorders, meningeal syndrome, and petechial rash),^61^ should be widely disseminated to primary care health workers. However, the diagnostic performance of each of the signs described in these campaigns, alone or in combination, is still insufficiently evaluated. Finally, timely care could be the focus of quality improvement strategies by optimizing children’s emergency referral and transport networks. For example, the creation of emergency telephone lines for health care professionals for specialized pediatric advice could improve the therapeutic timing. The development of mobile units specially trained to transport children with severe infection and the implementation of protocols for recognition and treatment of sepsis in each emergency department have shown their effectiveness in improving the quality of care and the outcome of children.^9,10,62,63^

### Limitations

Our study has some limitations. The generalizability of the results could be limited because it was conducted only in western France. The lack of systematic bacteriological examination of all children who died outside a PICU does not guarantee completeness of inclusions. A selection bias was also introduced by including only children who died or were hospitalized in a PICU. Indeed, rapid and effective care of certain children with an SBI may have led to a rapid improvement in the clinical condition and avoided admission to intensive care; this may have led to an overestimation of the frequency of suboptimal care and an underestimation of the association between the quality of care and outcome. The retrospective collection of the data regarding the children’s care pathway may have led to information bias and imprecision. For example, if vital signs (body temperature, blood pressure, and heart and breathing rates) or signs of severity were missing from the medical records, it was impossible to know whether this resulted from lack of recording of the information in the medical record or lack of adequate clinical examination. This situation could have distorted the experts’ assessment of the severity of the condition and its referral and, thus, the optimality of the care. The difficulty in accounting for the intrinsic severity of infection and individual characteristics of susceptibility to infection in each patient likely confounded the results of our analyses of the association between quality of care and outcome. Furthermore, because the quality of care received during PICU hospitalization was not assessed, we could not take it into account in studying the association between quality of care and outcome.

## Conclusions

By using a method adapted to the evaluation of quality of care, we showed that suboptimal management was frequent, occurring in approximately one-third of children with a COSBI, and was associated with the occurrence of severe sequelae. In caring for children with SBI, particular attention should be paid to the youngest children and those with comorbidities, and more attention should be paid to preventive strategies, such as vaccination, to reduce mortality. Medical care could probably be optimized by improving the preparedness of primary care physicians. Finally, specific public awareness campaigns to recognize the signs of sepsis are of paramount importance and should be implemented by health authorities.

## Notes

Supplement. **eFigure. **Directed Acyclic Graph Modeling the Relation Between the Quality of Initial Care and the Outcome of Children With a Community-Onset Severe Bacterial Infection **eAppendix. **Calculation of the Number of Patients to Be Recruited **eTable 1.** Degree of Agreement Among Experts on the Assessment of the Quality of Care According to the Kappa Coefficient Interpreted by the Landis and Koch Scale **eTable 2.** Detailed Characteristics of the Quality of Care Before Admission to a Pediatric Intensive Care Unit in Children With a Community-Onset Severe Bacterial Infection **eTable 3.** Examples of the Different Categories of Quality of Care Evaluated by the Experts for the Eight Major Types of Care
