# Supplementary material for: Prevalence, Characteristics, and Determinants of Suboptimal Care in the Initial Management of Community-Onset Severe Bacterial Infections in Children
Source: JAMA Netw Open. 2022 Jun 13;5(6):e2216778. doi: 10.1001/jamanetworkopen.2022.16778 (PMC9194668; doi:10.1001/jamanetworkopen.2022.16778)
Supplement: Supplement. — eFigure. Directed Acyclic Graph Modeling the Relation Between the Quality of Initial Care and the Outcome of Children With a Community-Onset Severe Bacterial Infection eAppendix. Calculation of the Number of Patients to Be Recruited eTable 1. Degree of Agreement Among Experts on the Assessment of the Quality of Care According to the Kappa Coefficient Interpreted by the Landis and Koch Scale eTable 2. Detailed Characteristics of the Quality of Care Before Admission to a Pediatric Intensive Care Unit in Children With a Community-Onset Severe Bacterial Infection eTable 3. Examples of the Different Categories of Quality of Care Evaluated by the Experts for the Eight Major Types of Care [file jamanetwopen-e2216778-s001.pdf]

Supplemental Online Content

Lorton F, Chalumeau M, Martinot A, et al. Prevalence, characteristics, and determinants of suboptimal care in the initial management of community-onset severe bacterial infections in children. *JAMA Netw Open*. 2022;5(6):e2216778. doi:10.1001/jamanetworkopen.2022.16778

eFigure. Directed Acyclic Graph Modeling the Relation Between the Quality of Initial Care and the Outcome of Children With a Community-Onset Severe Bacterial Infection
eAppendix. Calculation of the Number of Patients to Be Recruited
eTable 1. Degree of Agreement Among Experts on the Assessment of the Quality of Care According to the Kappa Coefficient Interpreted by the Landis and Koch Scale
eTable 2. Detailed Characteristics of the Quality of Care Before Admission to a Pediatric Intensive Care Unit in Children With a Community-Onset Severe Bacterial Infection
eTable 3. Examples of the Different Categories of Quality of Care Evaluated by the Experts for the Eight Major Types of Cares

This supplemental material has been provided by the authors to give readers additional information about their work.

© 2022 Lorton F et al. *JAMA Network Open.*

eFigure: Directed Acyclic Graph Modeling the Relation Between the Quality of Initial Care and the Outcome of Children With a Community-Onset Severe Bacterial Infection.

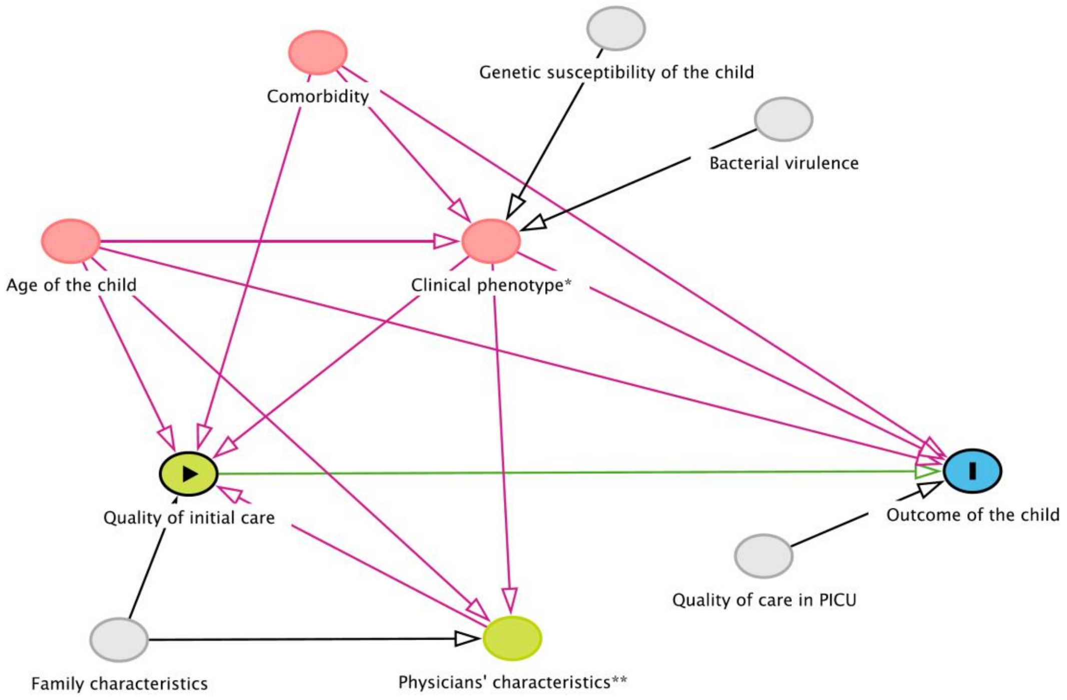

The green circle with a triangle corresponds to the exposure variable (the quality of initial care), the blue circle corresponds to the variable to be explained (the child's outcome: survival without sequelae, survival with severe sequelae at PICU discharge or death), the pink circles are the potential confounding factors (ancestors of the exposure variable and the variable to be explained), the green circle without a triangle is an "ancestor" of the exposure variable, and the grey circles are the variables unobserved.

*The clinical phenotype corresponds to the discharge diagnosis of the infection and its clinical severity, estimated by the presence of signs of severity at the first medical consultation.

**The characteristics of physicians correspond to the type of medical service consulted and the density of medical doctors in the home department.

© 2022 Lorton F et al. *JAMA Network Open.*

eAppendix: Calculation of the Number of Patients to Be Recruited.

The number of patients required based on the measure of the association between suboptimal management and occurrence of death in children with community-onset severe bacterial infection. Considering an expected percentage of suboptimal management of approximately 30% in surviving patients, with a risk α of 5% for a power of 80%, we needed to include at least 26 deceased patients and 153 surviving patients to be able to demonstrate an odds ratio ≥ 3.

© 2022 Lorton F et al. *JAMA Network Open.*

eTable 1: Degree of Agreement Among Experts on the Assessment of the Quality of Care According to the Kappa Coefficient Interpreted by the Landis and Koch Scale.[a]

	Kappa value +/- SD	Degree of agreement
**Global management**	0.67 ± 0.05	Substantial
**Time in seeking care by the family**	0.22 ± 0.04	Fair
**Medical evaluation of severity**	0.31 ± 0.05	Fair
**Patient referral**	0.40 ± 0.05	Fair
**Antibiotic therapy timing**	0.58 ± 0.12	Moderate
**Antibiotic therapy dosage**	0.26 ± 0.08	Fair
**Fluid bolus timing**	0.34 ± 0.15	Fair
**Fluid bolus volume**	0.41 ± 0.16	Moderate
**Assessment after fluid bolus**	0.36 ± 0.29	Fair

SD, standard deviation

[a]Landis JR, Koch GG. The measurement of observer agreement for categorical data. Biometrics.1977;33(1):159-174. Medline:843571 doi:10.2307/2529310

© 2022 Lorton F et al. *JAMA Network Open.*

eTable 2: Detailed Characteristics of the Quality of Care Before Admission to a Pediatric Intensive Care Unit in Children With a Community-Onset Severe Bacterial Infection

		Outcome of children, No. (%)		
	Total	Surviving without sequelae	Died	Surviving with sequelae
	N=259	N=207	N=27	N=25
**Global management, n§**	259	207	27	25
Certainly suboptimal	89 (34.4)	71 (34.3)	3 (11.1)	15 (60.0)
Possibly suboptimal	100 (38.6)	80 (38.6)	12 (44.4)	8 (32.0)
Optimal	70 (27.0)	56 (27.1)	12 (44.4)	2 (8.0)
Cannot judge	0 (0.0)	0 (0.0)	0 (0.0)	0 (0.0)
Not concerned	0	0	0	0
**Time in seeking care, n**	259	207	27	25
Certainly suboptimal	63 (24.3)	51 (24.6)	6 (22.2)	6 (24.0)
Possibly suboptimal	5 (1.9)	5 (2.4)	0 (0.0)	0 (0.0)
Optimal	184 (71.0)	144 (69.6)	21(77.8)	19 (76.0)
Cannot judge	7 (2.7)	7 (3.4)	0 (0.0)	0 (0.0)
Not concerned	0	0	0	0
**Evaluation of severity, n**	259	207	27	25
Certainly suboptimal	49 (18.9)	40 (19.3)	3 (11.1)	6 (24.0)
Possibly suboptimal	18 (6.9)	14 (6.8)	2 (7.4)	2 (8.0)
Optimal	186 (71.8)	149 (72.0)	20 (74.1)	17 (68.0)
Cannot judge	6 (2.3)	4 (1.9)	2 (7.4)	0
Not concerned	0	0	0	0
**Patient referral, n**	259	207	27	25
Certainly suboptimal	48 (18.5)	39 (18.8)	2 (7.4)	7 (28.0)
Possibly suboptimal	17 (6.6)	13 (6.3)	2 (7.4)	2 (8.0)
Optimal	183 (70.7)	147 (71.0)	21 (77.8)	15 (60.0)
Cannot judge	11 (4.2)	8 (3.9)	2 (7.4)	1 (4.0)
Not concerned	0	0	0	0
**Antibiotic therapy timing, n**	258	207	26	25
Certainly suboptimal	133 (51.6)	110 (53.1)	7 (26.9)	16 (64.0)
Possibly suboptimal	4 (1.6)	4 (1.9)	0 (0.0)	0 (0.0)
Optimal	120 (46.5)	93 (44.9)	18 (69.2)	9 (36.0)
Cannot judge	1 (0.4)	0 (0.0)	1 (3.8)	0 (0.0)
Not concerned	1	0	1	0
**Antibiotic therapy dosage, n**	233	190	22	21
Certainly suboptimal	25 (10.7)	22 (11.6)	1 (4.5)	2 (9.5)
Possibly suboptimal	1 (0.4)	1 (0.5)	0 (0.0)	0 (0.0)
Optimal	189 (81.1)	153 (80.5)	20 (90.9)	16 (76.2)
Cannot judge	18 (7.7)	14 (7.4)	1 (4.5)	3 (14.3)
Not concerned	26	17	5	4
**Fluid bolus timing, n**	230	187	22	21
Certainly suboptimal	128 (55.7)	110 (58.8)	6 (27.3)	12 (57.1)
Possibly suboptimal	5 (2.2)	5 (2.7)	0 (0.0)	0 (0.0)
Optimal	97 (42.2)	72 (38.5)	16 (72.7)	9 (42.9)
Cannot judge	0 (0.0)	0 (0.0)	0 (0.0)	0 (0.0)
Not concerned	29	20	5	4
**Fluid bolus volume, n**	169	131	22	16
Certainly suboptimal	56 (33.1)	49 (37.4)	3 (13.6)	4 (25.0)
Possibly suboptimal	3 (1.8)	3 (2.3)	0 (0.0)	0 (0.0)
Optimal	97(57.4)	68 (51.9)	18 (81.8)	11 (68.8)
Cannot judge	13 (7.7)	11 (8.4)	1 (4.5)	1 (6.2)
Not concerned	90	76	5	9
**Assessment after fluid bolus, n**	169	131	22	16
Certainly suboptimal	29 (17.2)	23 (17.6)	2 (9.1)	4 (25.0)
Possibly suboptimal	2 (1.2)	2 (1.5)	0 (0.0)	0 (0.0)

© 2022 Lorton F et al. *JAMA Network Open.*

Optimal	123 (72.8)	97 (74.0)	17 (77.3)	9 (56.3)
Cannot judge	15 (8.9)	9 (6.9)	3 (13.6)	3 (18.8)
Not concerned	90	76	5	9

N=number of children with community-onset severe bacterial infection.
§ The number "n" refers to children concerned by the care assessed.

© 2022 Lorton F et al. *JAMA Network Open.*

eTable 3: Examples of the Different Categories of Quality of Care Evaluated by the Experts for the Eight Major Types of Cares.

	Quality of care (as evaluated by the experts)		
	**Optimal**	**Certainly suboptimal**	**Possibly suboptimal***
**Time in seeking care by the family**	GP consultation at 2 hr of the onset of symptoms (fever, chills, pale skin, vomiting) in a 3-year-old child with no previous history	Medical consultation by telephone at 3 hr of the onset of a febrile purpuric rash with behavioral changes in a 22-month-old child	GP consultation at 12 hr of the onset of symptoms (fever, vomiting, headache, phono-photophobia) in a 15-year-old child with no previous history
**Physician's evaluation of severity**	GP's recognition of signs of sepsis in a 9-month-old child with fever, hypotonia, and tachycardia	Diagnosis of a viral infection and return home in a 14-year-old child with fever (temperature 41.3°C), vomiting, and headache. Vital signs not measured by the GP	Diagnosis of infant colic and return home in a 2-month-old infant with vomiting and grunting without fever
**Patient referral**	GP's referral to ED (transport <15 min) for suspected meningitis in a 5-year-old child	Discharge home with a diagnosis of otitis after ED consultation for fever, vomiting, and grunting with signs of sepsis at clinical examination in a 13-month-old child	Hospitalization of a 1-month-old infant with bacterial meningitis in a general pediatric ward
**Antibiotic therapy timing**	Antibiotic administration 30 min after the recognition of signs of sepsis	Antibiotic administration 5 hr after medical examination with signs of sepsis and suspicion of meningitidis	Antibiotic administration 2 hr after medical examination with signs of sepsis in a context of toxic skin eruption
**Antibiotic therapy dosage**	Ceftriaxone at 50 mg/kg/day in a context of sepsis with no source	Cefotaxime at 100 mg/kg/day in a context of bacterial meningitis	Amoxicillin/clavulanate at 80 mg/kg/day in a context of varicella with cellulitis[§]
**Fluid bolus timing**	20 min after the diagnosis of purpura fulminans	1 hr after the diagnosis of severe sepsis	40 min after the diagnosis of severe sepsis in a context of peritonitis
**Fluid bolus volume**	Volume at 20 ml/kg over 15 min in a context of purpura fulminans	Volume at 20 ml/kg over 1 hr in a context of purpura fulminans	Volume at 40 ml/kg over 1 hr for sepsis in a context of pneumonia with effusion
**Assessment after fluid bolus**	Clinical reassessment 10 min after the fluid bolus administration	No clinical reassessment after the fluid bolus administration	Clinical reassessment 1 hr after the fluid bolus administration

ED, emergency department; GP, general practitioner
* Children whose care was judged as "possibly suboptimal" were not included in the analyses of the association involving the quality of care.

© 2022 Lorton F et al. *JAMA Network Open.*

§ According to the French Pediatric Infectious Pathology Group, the recommended dosage of amoxicillin was 100 mg/kg/day at the time of the study (Moulin F, Quinet B, Raymond J, Gillet Y, Cohen R. Managing children skin and soft tissue infections [in French]. Arch Pediatr. 2008;15(suppl 2):S62-S67. Medline:19000857 doi:10.1016/S0929-693X(08)74218-0

© 2022 Lorton F et al. *JAMA Network Open.*
